# Supplementary material for: An Inhaled Nanoemulsion Encapsulating a Herbal Drug for Non-Small Cell Lung Cancer (NSCLC) Treatment
Source: Pharmaceutics. 2025 Apr 22;17(5):540. doi: 10.3390/pharmaceutics17050540 (PMC12115302; doi:10.3390/pharmaceutics17050540)

Supplementary Information

An Inhaled Nanoemulsion Encapsulating a Herbal Drug for Non-Small Cell Lung Cancer (NSCLC) Treatment

Mural Quadros, Mimansa Goyal, Gautam Chauhan, Dnyandev Gadhave and Vivek Gupta *

Department of Pharmaceutical Sciences, College of Pharmacy and Health Sciences, St. John's University, 8000 Utopia Parkway, Queens, NY 11439, USA; mural.quadros21@my.stjohns.edu (M.Q.); mimansa.goyal18@my.stjohns.edu (M.G.); gautam.chauhan16@my.stjohns.edu (G.C.); dnyanraj24@gmail.com (D.G.)

* Correspondence: guptav@stjohns.edu; Tel.: +1-718-990-3929

METHODS

Ultra-performance Liquid Chromatography (UPLC) quantification of Cela

The Waters Acquity series UPLC (Waters, Milford, MA, USA) system was used to quantify Cela. The stationary phase column was Xbridge BEH shield RP18 2.5 µm with a dimension of 3.0 × 100.0 mm. The binary mobile phase (MP) constituted 0.1% orthophosphoric acid and acetonitrile in a ratio of 15:85 v/v. The flow rate of MP was optimized to 0.5 mL/min. The eluents from the column were detected at wavelength λmax of 425 nm and a run time of 1.5 min. The resulting data was analyzed using Empower 3.0 software (Waters, Milford, MA, USA).

Transmission Electron Microscopy

Prior to scanning, the formulations were diluted with water, and 5 µL of each dilution was placed on the formvar-carbon-coated copper grid (100 mesh, Electron Microscopy Sciences, Hatfield, PA, USA). The grid was rinsed twice with Milli Q water and was stained with 2% uranyl acetate solution (Ladd Research Industries, Williston, VT, USA). Any excess solution was blotted out with Whatman 3 mm filter paper. The grid was air-dried and imaged at 20,000 × magnification using FEI Tecnai Spirit TWIN TEM (FEI, Hillsboro, OR, USA) operated at 120 kV voltage.

In-vitro Aerosolization

The NGI was cooled in the refrigerator at 4 °C for 90 min to aid condensation of the vapors onto the cooled NGI plates. The formulation, NE-Cela, was diluted 50 times with miliQ water, and 2 mL was placed into a PARI LC PLUS® nebulizer cup, which was then drawn through the NGI with the help of a vacuum pump (Copley Scientific, UK) operated at 15 L/min for 8 mins. Samples were collected and examined for Cela content at each stage, i.e., Stages 1–8, including cup (device), induction port (mouth), and throat, using the developed UPLC method. Mass median aerodynamic diameter (MMAD, µm) and geometric standard deviation (GSD, µm) are important

parameters describing the inhalable capability of the NE. The MMAD and GSD were calculated by quantifying the drug content at each stage of the NGI using log probability analysis. Fine particle fraction (FPF, %) was also determined as the fraction of the emitted dose deposited in the NGI.

Cytotoxicity Study

Briefly, cells were grown in FBS-supplemented RPMI-1640 media, and 2.5 × 103 cells/well were seeded in a 96-well plate and subsequently incubated overnight for 37 °C/5% CO2 to aid cell attachment. The cells were treated with NE-Cela and Cela over a concentration range of 0.16 to 5 mM for 48 h. Following this period, the treatments were replaced with MTT solution and reincubated for 2 h at 37 °C/5% CO2. Later, DMSO was added to dissolve the formazan crystals. The cytotoxicity of the treatments was determined by measuring absorbance 570 nm using a TECAN plate reader (Tecan Group Ltd., Männedorf, Switzerland). The absorbance of treatments was compared against the control to calculate the % cell viability.

Wound Healing Assay

Briefly, 1.0 × 10^5 A549 cells/well were plated in a 24-well plate and then incubated overnight at 37 °C/5% CO_2 to aid the formation of a confluent cell monolayer. The following day, a scratch was made along the center of each well using a 100 μL sterile pipette tip. All wells were washed thrice with PBS to remove the cells that were detached from scratch. The scratched wells were imaged using an inverted microscope (LAXCO, Mill Creek, WA, USA) with a 10X magnification objective at 0, 24, and 48 h. The images at time 0 h were scratched after scratch formation but before the addition of the treatments. The cells were treated with NE-Cela or Cela at two concentrations (1.2 μM and 0.6 μM) while wells with only media, i.e., no treatment, served as

control. Cell migration and % scratch closure were observed using Image J software to assess the inhibitory effect of NE-Cela and Cela.

Clonogenic Assay

A549 cells (250 cells/mL) were seeded in a 6-well plate with volume of 1 mL in each well. The plates were incubated overnight to allow adherence of cells to the plate. The following day, the media was removed and replaced with treatments containing Cela and NE-Cela at 1.2- and 0.6-μM concentrations. The plate was incubated for 48 h and replaced every alternate day with treatments and media (control) for 7 days. After 7 days, all media were removed and stained with crystal violet. This was done by washing the wells twice with ice-cold PBS and then fixing the cells with 4% PFA solution for 10 min. The fixed cells were rewashed with ice-cold PBS and stained overnight with 0.01% (w/v) crystal violet solution. The next day, the stain was washed with distilled water, and the images of all wells were captured with a digital camera. Colony counter software (Open CFU) counted the stained colonies digitally.

Spheroid Assay

A549 cells were seeded at 5.0×10^2 cells/well density in ultra-low attachment 96-well U bottom plates (Nuclon® sphere, Thermo-Fisher Scientific, Waltham, MA, USA). Once seeded, the plates were incubated at 37 °C/5% CO_2 for spheroid formation until 72 h. Post incubation, the cells were treated in two different regimens, i.e., single dose and multidose, and at two different concentrations, i.e., at an IC_{50} value of 1.2 μM and half IC_{50} value of 0.6 μM. Single-dose treatment was provided only once at the beginning of the experiment, while multidose treatment involved dosing at an interval of every 72 h until the termination of the experiment. This was done by carefully replacing only half a volume of media with treatments to avoid aspiration of tumor spheroids. Treatment efficacy was evaluated by imaging the spheroids using optical microscopy

by LMI-6000 inverted microscope (LAXCO, Mill Creek, WA, USA) every 72 h. Spheroid characteristics such as spheroid diameter and volume using Image J software.

Live-Dead Assay

The kit comprised two dyes: calcein AM, which stains live cells, and EthD-III, which stains dead cells. The media was replaced with 200 µL of reagents as instructed in the kit protocol. The plate was incubated with reagents in the dark for 30 min and was later imaged using a fluorescence microscope EVOS FL (Thermo Fisher Scientific, Waltham, MA, USA).

Stability Studies

The stability of NE-Cela over a 28-day period was evaluated at two storage conditions: 4°C and 40°C with 75% relative humidity (RH).

Fig. S1: **(A)** Overlay chromatograms of developed UPLC method for Quantification of Cela, **(B)** standard curve ranging 1-100 ppm, with fitted equation.

A)

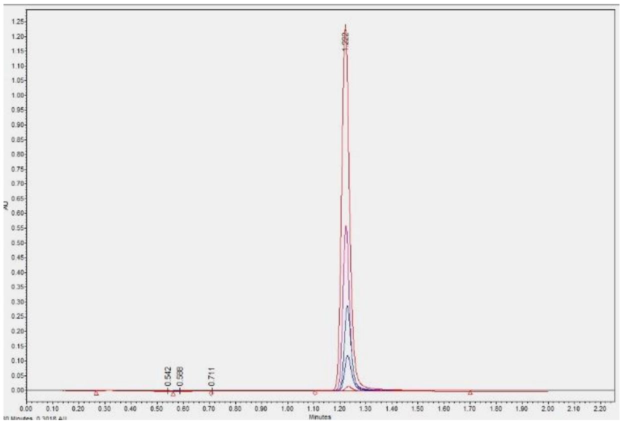

B)

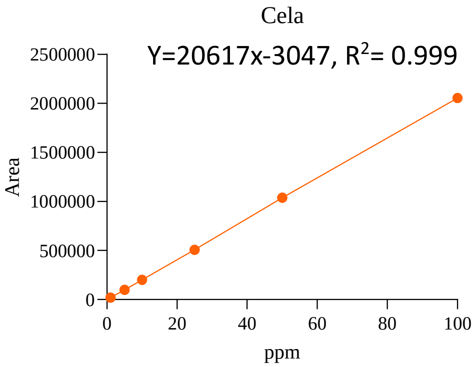

Fig. S2: Drug release fitting kinetic various models.

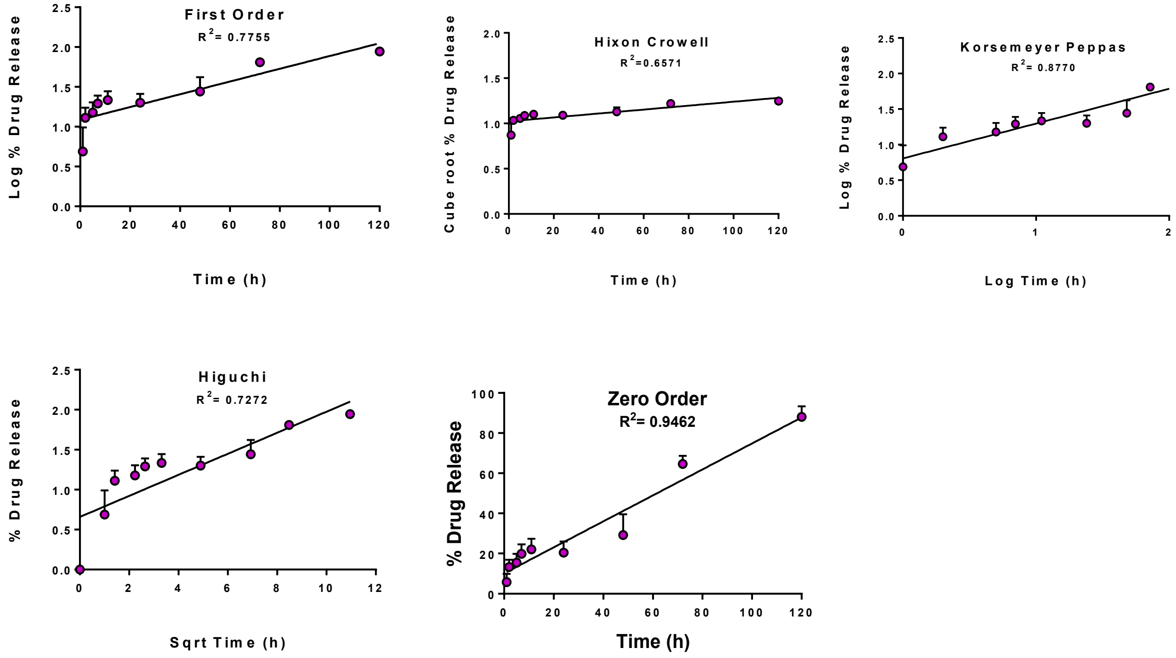

Fig. S3: Representative images demonstrating the effect of treatment (1.2 μM) on spheroids on 15 days of treatment using a live-dead cell assay kit. Florescent images of the spheroids were obtained using fluorescence microscopy. Viable cells are represented by green fluorescence, while red fluorescence indicates dead cells in spheroids.

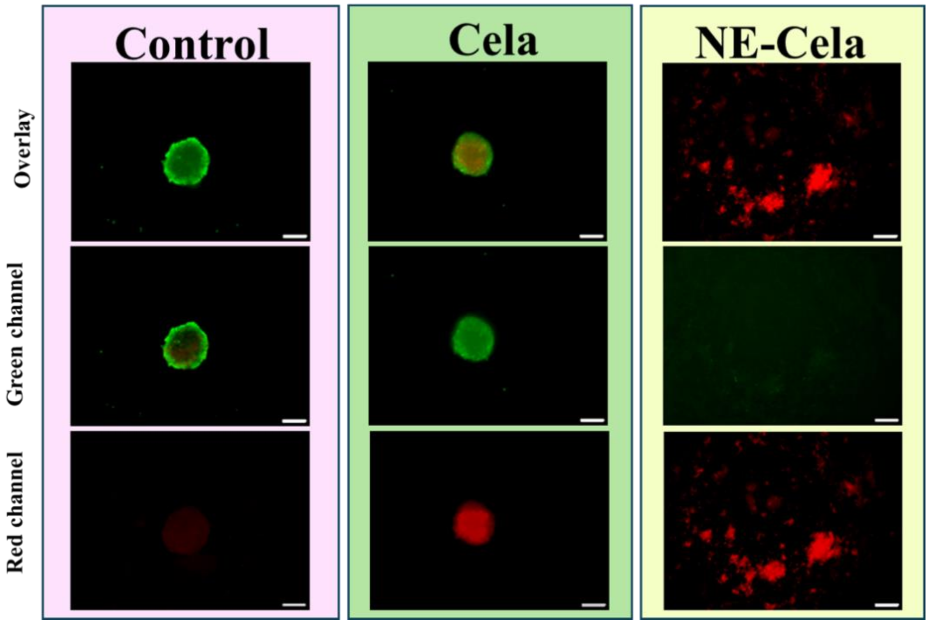

Fig. S4: Stability analysis for NE-Cela at 4°C & 40°C and 75% RH over a period of 4 weeks. The characteristics analyzed were **(A)** % Drug entrapment, **(B)** Globule size (nm), **(C)** Polydispersity index, and **(D)** Zeta potential (mV).

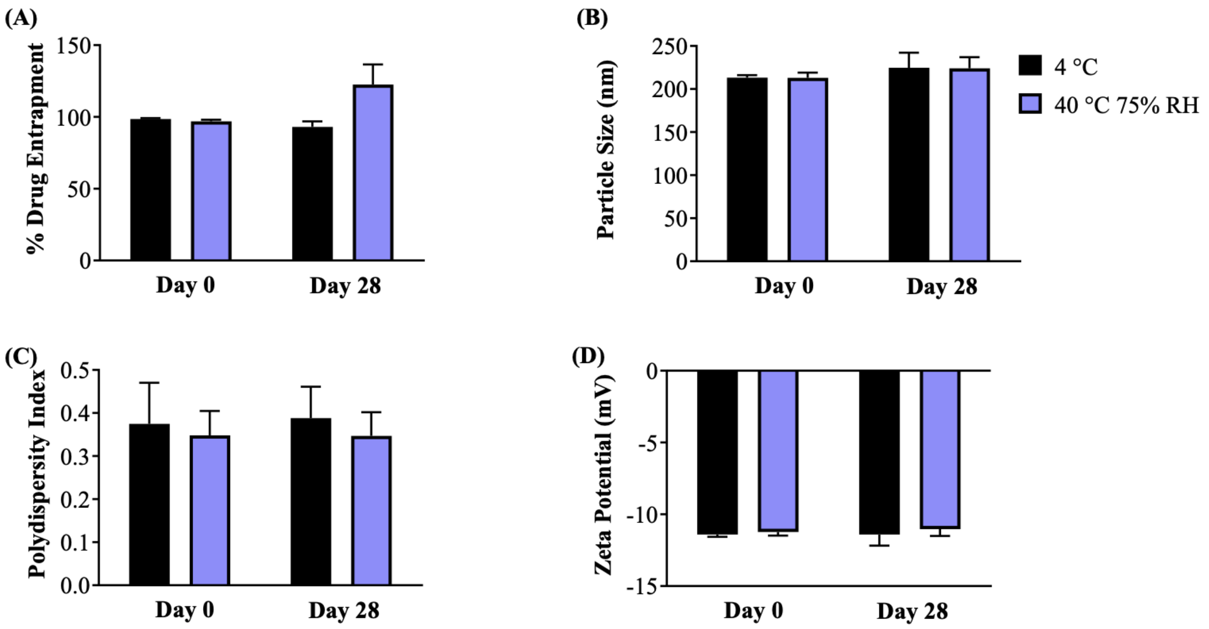

Supplement: Supplementary file 1 [file pharmaceutics-17-00540-s001.zip › pharmaceutics-3569238-supplementary.pdf]
